# Zedoarondiol inhibits human bronchial smooth muscle cell proliferation through the CAV-1/PDGF signalling pathway

**DOI:** 10.1038/s41598-024-63970-4

**Published:** 2024-06-07

**Authors:** Yinglan Lyu, Wandi Feng, Jingze Song, Chunguo Wang, Yu Fu, Baosheng Zhao, Yanyan Meng

**Affiliations:** 1 College of Chinese Medicine, Beijing University of Chinese Medicine, Beijing, 100029 China; 2 Institute of Materia Medica, Chinese Academy of Medical Sciences and Peking Union Medical College, Beijing, 100050 China; 3 The Third Affiliated Hospital of Beijing University of Chinese Medicine, Beijing, 100029 China; 4 Beijing Research Institute of Chinese Medicine, Beijing University of Chinese Medicine, 11 North 3Rd Ring Eastern Road, Beijing, 100029 China

**Keywords:** Cell biology, Drug discovery, Molecular biology, Biomarkers, Diseases, Health care, Medical research, Molecular medicine, Pathogenesis

## Abstract

Airway remodelling in lung diseases can be treated by inhibiting excessive smooth muscle cell proliferation. Zedoarondiol (Zed) is a natural compound isolated from the Chinese herb *Curcuma longa*. The caveolin-1 (CAV-1) is widely expressed in lung cells and plays a key role in platelet-derived growth factor (PDGF) signalling and cell proliferation. This study aims to investigate the effect of Zed on human bronchial smooth muscle cell (HBSMC) proliferation and explore its potential molecular mechanisms. We assessed the effect of Zed on the proliferation of PDGF-stimulated HBSMCs and performed proteomic analysis to identify potential molecular targets and pathways. *CAV1* siRNA was used to validate our findings in vitro. In PDGF-stimulated HBSMCs, Zed significantly inhibited excessive proliferation of HBSMCs. Proteomic analysis of zedoarondiol-treated HBSMCs revealed significant enrichment of differentially expressed proteins in cell proliferation-related pathways and biological processes. Zed inhibition of HBSMC proliferation was associated with upregulation of *CAV1*, regulation of the CAV-1/PDGF pathway and inhibition of MAPK and PI3K/AKT signalling pathway activation. Treatment of HBSMCs with *CAV1* siRNA partly reversed the inhibitory effect of Zed on HBSMC proliferation. Thus, this study reveals that zedoarondiol potently inhibits HBSMC proliferation by upregulating CAV-1 expression, highlighting its potential value in airway remodelling and related diseases.

## Introduction

Airway smooth muscle cells (ASMCs) are effectors of proliferation and remodelling in many chronic airway diseases (including asthma, chronic obstructive pulmonary disease, etc.), and airway smooth muscle (ASM) response is an important indicator of airway remodelling and asthma severity^1,2^. Airway smooth muscle cells (ASMCs) not only receive various signals via receptors and secrete various signalling molecules to act on other downstream target cells but also feedback to upstream pathways or receive feedback from downstream pathways, forming an extremely complex network^3,4^. ASMCs play an important role in airway structure and function by modulating airway inflammation through the release of pro- and anti-inflammatory mediators and immunomodulatory factors. In addition, ASMCs can cause hyperproliferation or participate in airway remodelling by producing and regulating extracellular matrix, leading to bronchial lumen narrowing, airway hyperresponsiveness and progressive loss of lung function^5,6^. A variety of external stimuli (e.g., PDGF) and extracellular matrix components regulate the phenotypic plasticity of airway smooth muscle, which signals through a network of intracellular cascades to control the transcription and translation of smooth muscle-specific genes^7,8^. To this end, clarifying the factors and related molecular mechanisms of ASMC proliferation is an important strategy to prevent and alleviate airway remodelling in the context of airway-related diseases.

Caveolae are spongy invaginations of the lipid membrane that exist in various types of cells. Caveolae contain a large amount of caveolin, which is a regulatory centre for transmembrane signal transduction^8,9^. Three types of caveolin from different genes have been identified (caveolin-1, caveolin-2, and caveolin-3). Caveolin-1 (Cav-1) is the main structural and functional protein of caveolae^10–12^. CAV-1 plays a crucial role in pulmonary dysfunction by functioning as both a regulatory and scaffolding protein in various signalling cascade protein complexes within the lungs^8^. PDGF-BB is an important mitogen, which can stimulate the division and proliferation of vascular smooth muscle cells, fibroblasts, glial cells and other cells, and has a regulatory effect on ontogeny and cell differentiation. PDGFR-β is the receptor for PDGF-BB. It has been well documented that CAV-1 plays an important role in regulating PDGF-induced signal transduction and cell proliferation, and PDGFR-β has been reported to be closely related to CAV-1 in cell signalling^13^. CAV-1 can inhibit cell proliferation caused by increased activity of enzymes such as p42/p44 MAPK, PI3K, and AKT in signal transduction pathways and play a role in regulating proliferation and the cell cycle^14–16^. Therefore, regulating the CAV-1/PDGF pathway is an important process to maintain ASMC quiescence.

**Figure 1:**
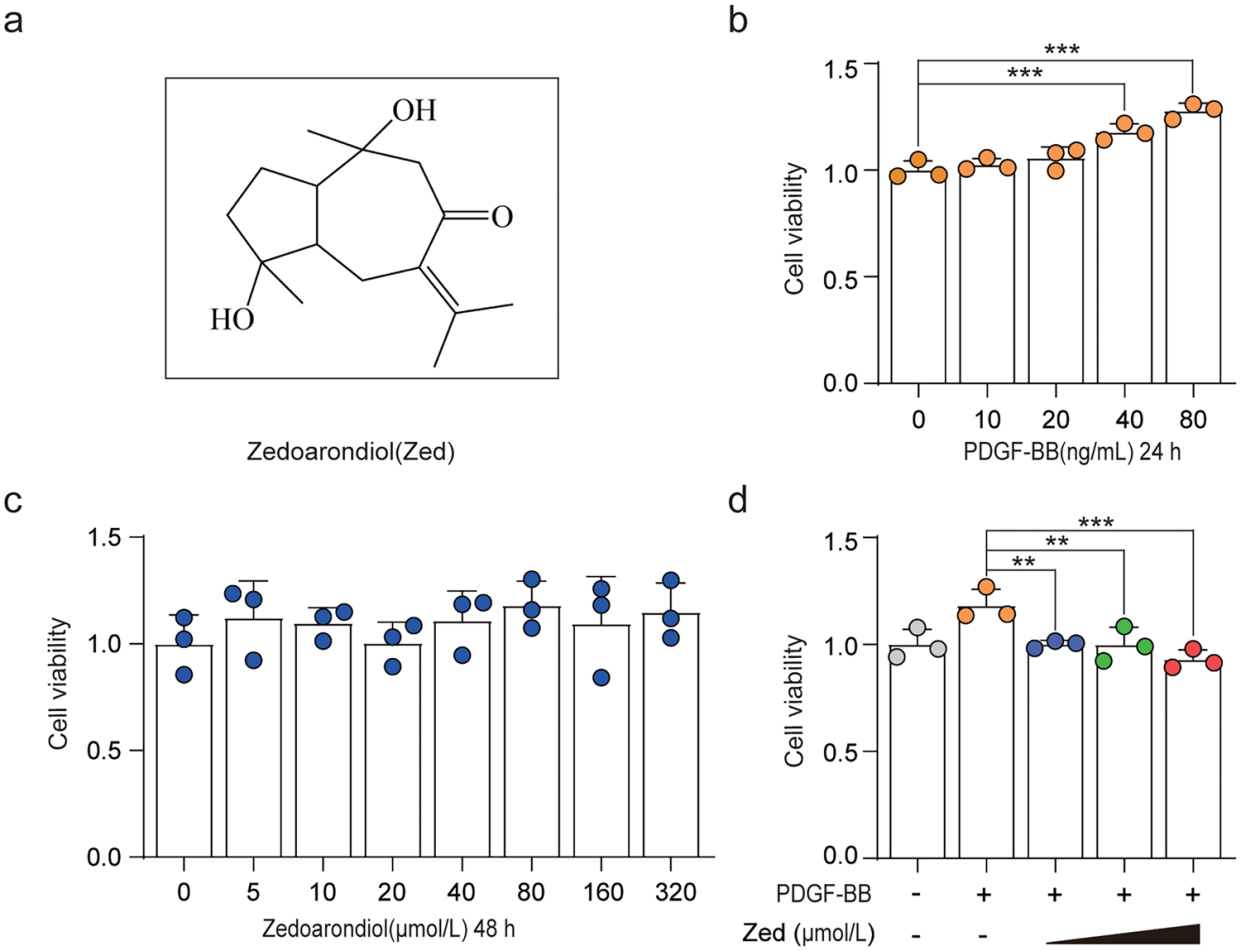
Zedoarondiol inhibits the proliferation of HBSMCs induced by PDGF-BB. (**a**) Zedoarondiol (Zed) structure. (**b**) Serum starved HBSMCs were treated with PDGF-BB (10–80 ng/mL, 24 h), and then the cell proliferation was determined using a CCK-8 assay. (**c**) Serum starved HBSMCs were treated with Zed (5–320 µmol/L, 24 h), and then the cytotoxicity of the compound was determined using a CCK-8 assay. (**d**) Serum starved HBSMCs were pretreated with Zed (20, 40, 80 µmol/L) for 24 h and then co-treated with PDGF-BB (final concentration 80 ng/mL) and Zed (20, 40, 80 µmol/L) for another 24 h. The cell viability was tested using a CCK-8 assay. Data shown as mean ± SD. *P < 0.05, **P < 0.01, ***P < 0.001.

*Zedoariae rhizoma* (*Curcuma zedaria* Roscoe, e’zhu in Chinese) is a Chinese herb belonging to the Zingiberaceae family. The main bioactive components of the *Zedoariae rhizome* include curcumin and elemene, which have a wide spectrum of pharmacological activities, including anti-inflammatory, antioxidant, antitumour and antiproliferative properties^17,18^. Zedoarondiol is a sesquiterpene lactone (Fig. 1a), which is the major compound isolated from *Zedoariae rhizome.* Its chemical structure and properties are different from those of curcumin and elemene. It is a new type of curcuma guaiane sesquiterpene compound and with a patent license. Previous studies have shown that zedoarondiol (Zed) can regulate the proliferation of vascular smooth muscle cells (VSMCs) through CAV-1-mediated endothelial nitric oxide synthase and can also inhibit the proliferation of VSMCs induced by PDGF-BB through AMPK signalling pathways^19^. However, it is unclear whether Zed regulates the proliferation of HBSMCs by regulating CAV-1 expression. In our study, PDGF-BB was used to establish an HBSMC proliferation model to explore the effects of Zed on proliferation and the cell cycle. Based on the CAV-1/PDGF pathway, the molecular mechanism by which Zed inhibits the proliferation of HBSMCs was elucidated, providing experimental evidence for the clinical application of Zed.

## Materials and methods

### HBSMC culture

Human bronchial smooth muscle cells (HBSMCs) were obtained from Procell Life Science and Technology Co., Ltd (Wuhan, China) and cultured on DMEM that contained 10% foetal bovine serum and 1% penicillin at 37 °C in humidified atmospheres with 5% CO_2_.

### Proliferation and viability assays

To explore the optimal concentration of HBSMCs induced by PDGF-BB, HBSMCs were inoculated into 96-well plates overnight at a rate of 1 × 10^4^ cells/well, and serum-free DMEM F12 medium was added to the plates for starvation treatment for 24 h to synchronize the cells. Then, PDGF-BB was added at final concentrations of 10, 20, 40 and 80 ng/mL. To test the effect of Zed on the proliferative activity of normal HBSMCs, Zed solution was added to the medium of HBSMCs, and the final concentrations were 5, 10, 20, 40, 80, 160, and 320 µmol/L. To test the effect of Zed on the proliferative activity of HBSMCs induced by PDGF-BB, cells were pretreated with Zed for 24 h and then cotreated with PDGF (final concentration 80 ng/mL) and Zed for another 24 h. Viability was assessed via CCK-8 assays following the manufacturer's instructions.

### Flow cytometric analyses

Serum-starved HBSMCs were pretreated with Zed for 24 h and then cotreated with PDGF and Zed for another 24 h. Apoptosis was analysed using an Annexin V-FITC Apoptosis Detection Kit according to the manufacturer's instructions (BD Pharmingen, San Diego, USA). Stained cells (a total of at least 10,000 events) were analysed with a BD LSR Fortessa flow cytometer (BD, San Diego, USA).

HBSMCs were grown and treated as described above. After the cells were collected, they were fixed in 70% ethanol and stored for 24 h at −20 °C. Cell pellets were centrifuged and treated with 500 µL of PI staining buffer (containing 200 mg/mL RNase A and 50 mg/mL PI) for 15–30 min at room temperature after centrifugation. In flow cytometry, fluorescent signals were measured using the PE channel after recording 10,000 events for each sample. Data analysis was performed using FlowJo software.

### Protein extraction and quantification

HBSMC protein lysates were prepared using RIPA lysis buffer and protease and phosphatase inhibitors. The protein concentration was determined using a BCA protein assay. After determination of the concentration, each group of proteins was quantified as 8 µg/µL. The peptides were acidified with trifluoroacetic acid at a concentration of 10% to a final concentration of 0.4%, the reaction was terminated, and the liquid was collected; the liquid was added to 200 mL liquid phase vials and subjected to examination.

### NanoLC-Orbitrap Fusion Lumos MS data acquisition method

The protein samples were detected by liquid chromatography-tandem mass spectrometry using an Acclaim PepMap 100C18 liquid chromatography column (NanoLC-Orbitrap Fusion Lumos MS). Data acquisition was combined with data-dependent mode scanning and scanning using data-independent (DIA) mode. The spectra were obtained via XCalibur 4.1 software, and the information of each data point was statistically analysed.

### Statistical analysis and processing of data

The obtained data were searched with ProteomeDiscoverer 2.4, and the human canonical dataset of the UniProtKB/SwissProt database was used to build the QC library. The library was imported into Shyline software for statistical analysis.

### Transfection of siRNAs

*CAV1* siRNA (AGACGAGCUGAGCGAGAAGCATT) was purchased from Weichuangbojing Biotech (Beijing, China). The scrambled siRNA sequenced used for *CAV1* knockdown are shown in the supplementary file (Table 1). A 50 nM dose of siRNA was transfected into HBSMCs grown to 50% confluence in six-well plates. A nonsense, scrambled siRNA was used as a negative control for transfection. The cells were transfected with 50 nM siRNA in FCS-free medium for 6 h, followed by 48 h of culture at 37 °C and 5% CO_2_. *CAV1* knockdown efficiency was evaluated 48 h post transfection by qRT‒PCR or western blotting.

### RNA isolation and real-time quantitative (q) PCR

Cellular RNA was extracted using TRIzol reagent (Invitrogen, Carlsbad, CA, USA). A PrimeScript RT reagent kit (Accurate, Human, China) was used to synthesize cDNA, and SYBR Green Master Mix (Beyotime Biotechnology) was used to carry out qRT‒PCR. The sequences of primers used for qPCR are as follows Table 1.

**Table 1:** Primer sequences for qPCR.

Primer	Sequence (5′–3′)
ACTIN	
Forward	CATGTACGTTGCTATCCAGGC
Reverse	CTCCTTAATGTCACGCACGAT
CAV1	
Forward	CATCCCGATGGCACTCATCTG
Reverse	TGCACTGAATCTCAATCAGGAAG

### Western blot analysis

As previously described^20^, protein samples were separated by SDS‒PAGE electrophoresis and then transferred to PVDF membranes. The membranes were then blocked with 5% skim milk and shaken at room temperature for 1.5 h. After overnight incubation with primary antibodies at 4 °C, the membranes were incubated with the corresponding secondary antibodies for 1 h at room temperature and exposed and photographed in a sensitive multifunctional imager. Antibody suppliers, catalog number and molecular weight are shown in the supplementary file (Table 2).

### Statistical methods

Data are expressed as the mean ± standard deviation. Multiple group comparisons were conducted using one-way ANOVA followed by the LSD test (data with homogeneous variance) in SPSS. *P* < 0.05 was considered to indicate statistical significance.

## Results

### Zedoarondiol inhibits the PDGF-BB-induced proliferation of HBSMCs

Zed has the molecular formula of C_15_H_24_O_3_, as shown in Fig. 1a. To explore the optimal concentration of PDGF-BB for inducing the proliferation of HBSMCs, HBSMCs were stimulated with different concentrations of PDGF-BB (10, 20, 40, and 80 ng/mL) for 24 h, and cell viability was determined using a CCK-8 assay. As shown in Fig. 1b, PDGF-BB concentrations of 40 ng/mL and 80 ng/mL promoted the proliferation of HBSMCs. To ensure the stability of the model group, an 80 ng/mL PDGF-BB concentration was selected in the subsequent experiment. A CCK-8 test was used to evaluate the cytotoxic effect of Zed treatment at different concentrations for 48 h on HBSMC survival. As shown in Fig. 1c, Zed treatment below 320 µmol/L did not affect the viability of HBSMCs (untreated control cells were used as a reference). Then, we selected concentrations of 20, 40, and 80 µmol/L to assess Zed’s effect on PDGF-BB-induced HBSMC proliferation. The results showed that HBSMC proliferation was promoted by PDGF‐BB, while the promotive effect was suppressed by Zed (Fig. 1d).

### Zedoarondiol causes HBSMC arrest in ghd G1 phase and induces apoptosis

**Figure 2:**
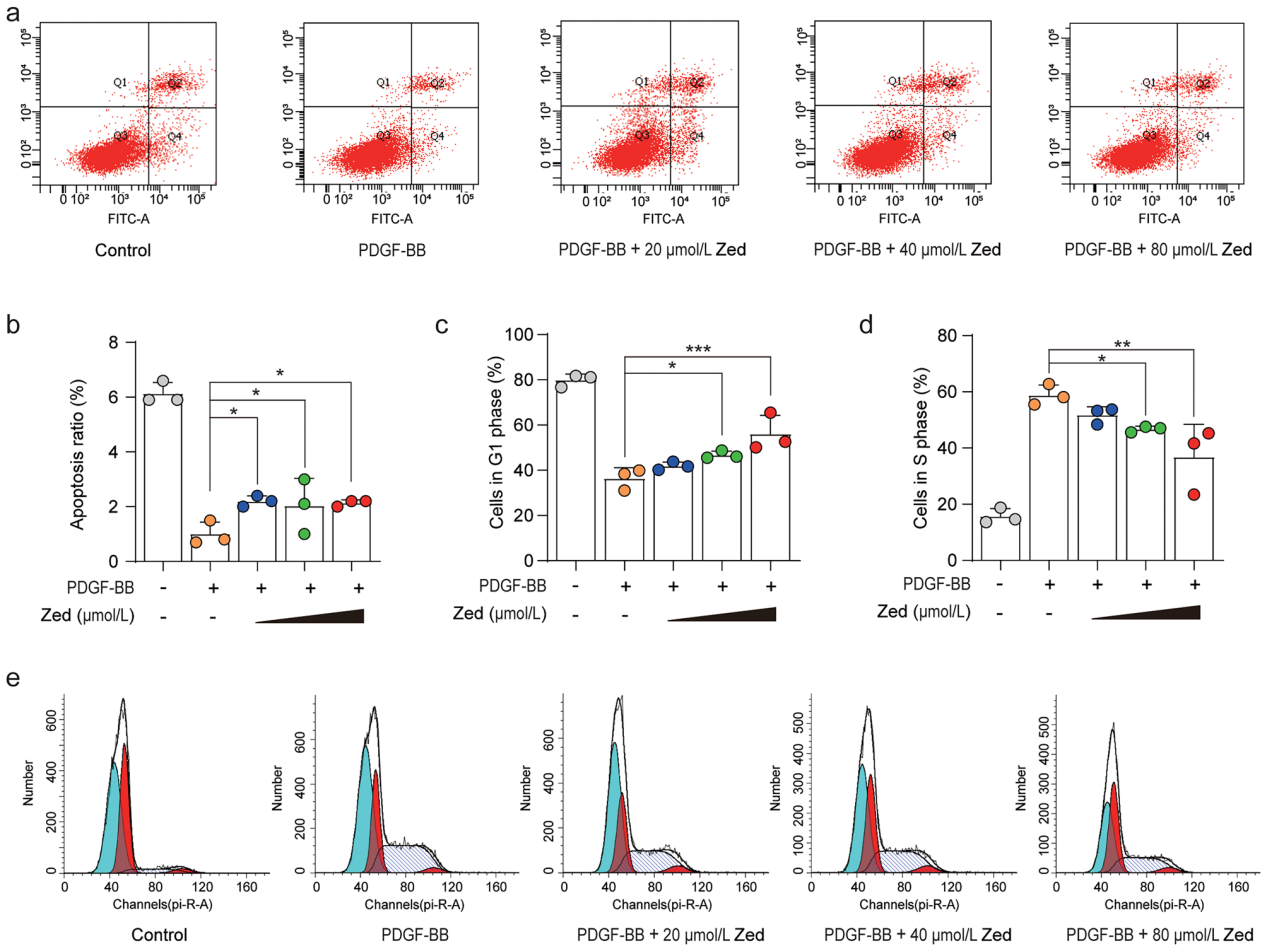
Zedoarondiol induces cell apoptosis in HBSMCs and blocks proliferation of HBSMCs induced by PDGF-BB in G1 phase. (**a**) HBSMCs were treated as in Fig. 1d. The apoptosis of HBSMCs was evaluated by annexin V/PI staining and flow cytometry. (**b**) Quantitative analysis of cell apoptosis. Apoptosis ratio = Q2 + Q4. (**c**, **d**) Quantitative analysis of cell cycle distribution in (**e**) HBSMCs. G1 phase: the first peak in (**e**) HBSMCs; S phase: the gently spanning peak in (**e**) HBSMCs. (**e**) HBSMCs were treated as in Fig. 1d. The PI signal was detected from the PE channel in flow cytometry. Data shown as mean ± SD. *P < 0.05, **P < 0.01, ***P < 0.001.

To investigate whether Zed-induced inhibition of proliferation was mediated by cell death, we further evaluated the effects of Zed on cell apoptosis in HBSMCs using annexin V/PI double staining (Fig. 2a,b). The PI-negative and Annexin V-positive cells were considered early apoptotic cells, while cells positive for both signals were considered late apoptotic cells. Total apoptotic cells were quantified by considering both early and late apoptotic cells. We found that after PDGF-BB treatment, the apoptosis rate was significantly reduced. Zed treatment caused an increase in the percentage of apoptotic cells, which indicated that the effect of Zed on PDGF-BB-induced HBSMC proliferation may be related to induction of HBSMC apoptosis.

The progression of the cell cycle is also closely linked to cell proliferation. Thus, a further investigation was conducted with PI staining and flow cytometry to determine whether Zed affects cell cycle progression. As shown in Fig. 2c–e, the fraction of HBSMCs in the G1 phase of the cell cycle decreased from 79.84 to 36.41% after PDGF-BB treatment, whereas the proportion of HBSMCs in the S phase of the cell cycle increased from 15.69 to 58.78%. Zedoarondiol interventions of 20 and 40 µmol/L did not show a significant difference in cell cycle distribution. We found that 80 µmol/L Zed treatment increased the proportion of HBSMCs in the G1 phase to 56.08% and reduced the proportion of HBSMCs in S phase to 36.75%. Therefore, these results suggested that Zed can effectively block PDGF-BB-induced HBSMCs in the G1 phase and prevent them from entering S phase.

### Quantitative proteomic analysis of the action of Zed on PDGF-BB-induced proliferation in HBSMCs

**Figure 3:**
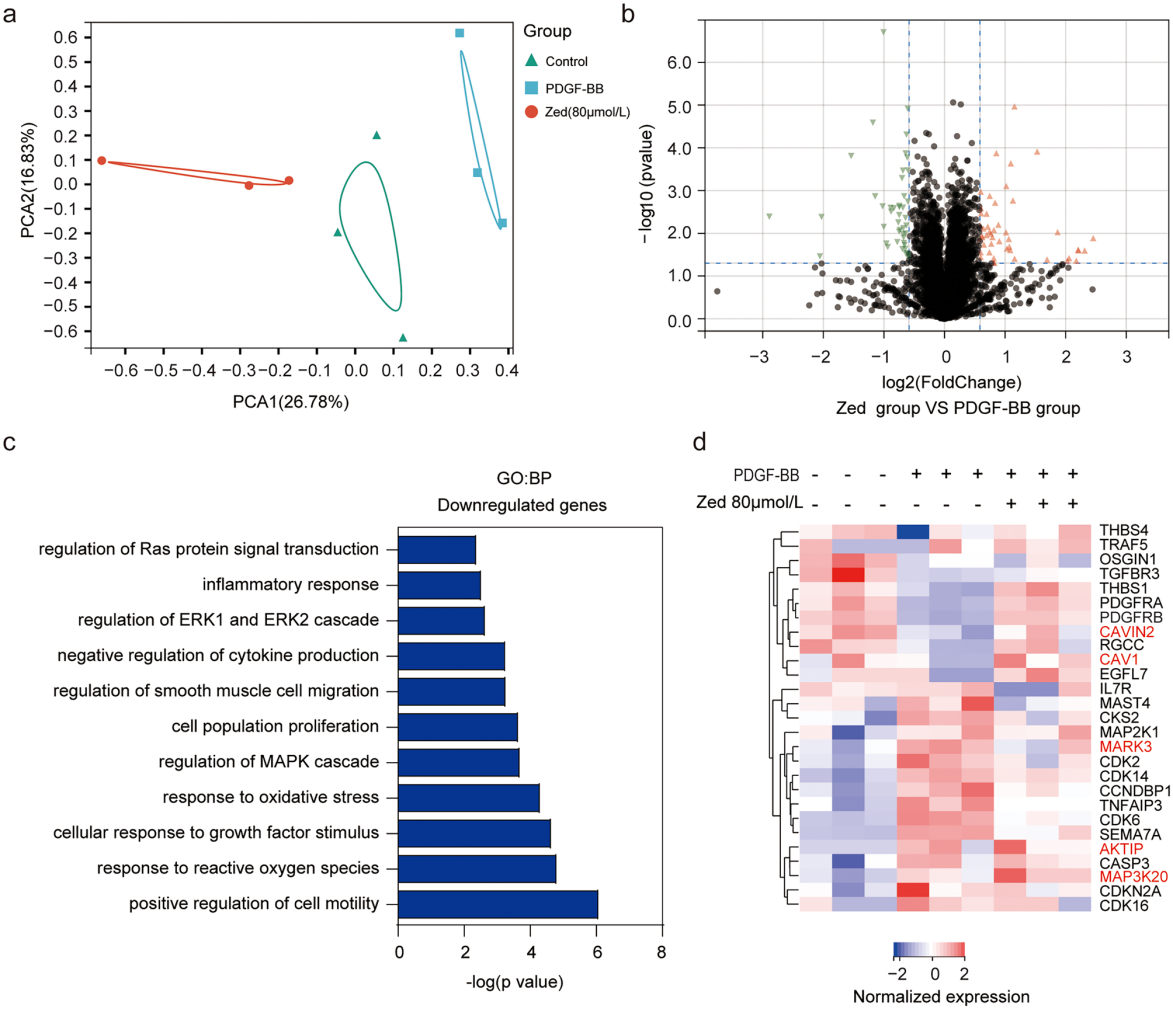
Quantitative proteomic analysis reveals the pathways enriched for the significantly changed proteins that mediate the inhibition effect of zedoarondiol on proliferation. (**a**) HBSMCs were treated as in Fig. 1d. Multivariate analysis by means of principal component analysis (PCA) of all group specific protein concentration levels allowed clear separation of the different samples according to their treatment group. (**b**) Volcano plot displays the distribution of proteins (*P* < 0.05, log_2_(fold change) > log_2_(1.5)) quantified in proteomic study. (**c**) GO pathway enrichment analysis (BP: biological process) based on significantly changed proteins. Representative pathways are shown. (**d**) Heatmap shows the normalized abundances of significantly changed proteins (affect cell proliferation) (*P* < 0.05, log_2_ (fold change) > log_2_ (1.5)) in Zed-treated cells compared with the PDGF-BB-stimulated cells.

To determine how Zed affects the proliferation of HBSMCs induced by PDGF-BB and its underlying molecular targets, a proteomic study was carried out on PDGF-BB-stimulated HBSMCs with or without Zed treatment. The PCA results showed distinct clustering of the individual samples according to their treatment group (Fig. 3a). As shown in the volcano plot (Fig. 3b), a total of 7971 differentially expressed proteins were identified between the Zed- and control-treated groups under PDGF-BB stimulation; the results were filtered to obtain 55 upregulated proteins and 51 downregulated proteins. Moreover, GO biological process (GO: BP) analysis was performed with the differentially expressed proteins and revealed enrichment of cell proliferation-related pathways and biological processes, such as the MAPK signalling pathway, response to growth factor stimulus, and inflammatory response (Fig. 3c). The relative expression levels of proteins are presented as a heatmap in Fig. 3d. We thus focused on the expression of key proteins involved in cell proliferation, such as CAV-1/PDGFRB, MARK3, MAP3K20, and AKTIP, and observed that these proteins were obviously upregulated with Zed treatment. MARK3, MAP3K20, and AKTIP involved in the MAPK signalling pathway and PI3K-AKT signalling pathway were significantly downregulated by Zed (Fig. 3d). Previous reports have indicated that CAV-1 inhibits the proliferation of ASMCs by binding to PDGFR and inhibiting the interaction of the PDGF-PDGFR axis and activation of its downstream signalling pathway^14^. These observations suggest that CAV-1//PDGF and MAPK, PI3K/AKT signalling pathway might play critical roles in the inhibitory effect of Zed on cell proliferation.

### Zedoarondiol upregulates CAV-1 expression and increases the colocalization of CAV-1 and PDGFRβ in HBSMCs

**Figure 4:**
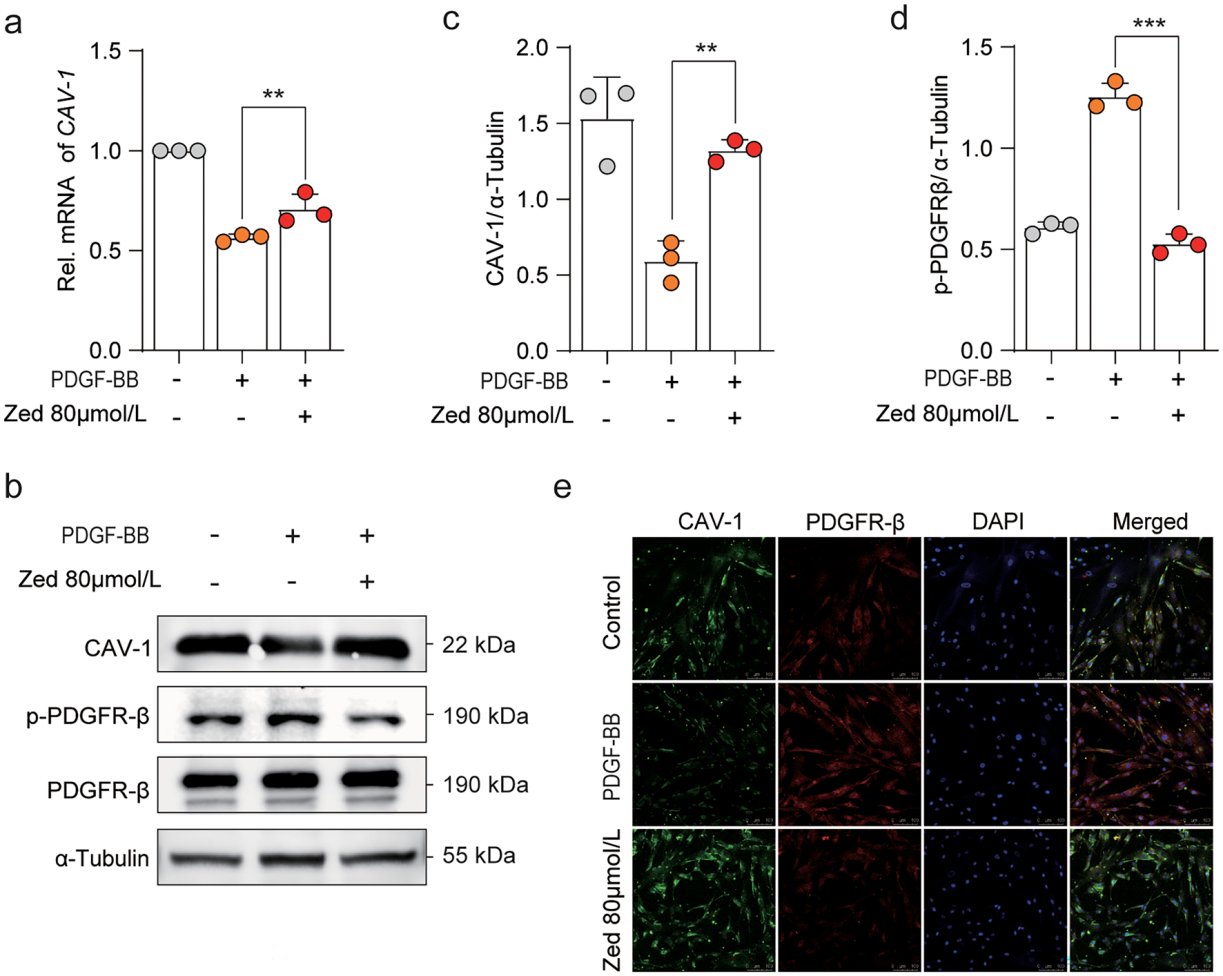
Zedoarondiol regulates CAV-1/PDGFRβ binding in HBSMCs cells. (**a**) qPCR analysis of *CAV1* mRNA expression in HBSMCs treated as described in Fig. 1d. (n = 3 per group). (**b**) Western blot analysis of CAV-1、p-PDGFR-β and PDGFR-β in HBSMCs. HBSMCs were treated as in Fig. 1d. (**c**, **d**) Quantitative analysis of protein expression was shown. (**e**) The colocalization of CAV-1 (green) and PDGFR-β (red) in HBSMCs treated as in (**a**) (n = 3 per group). Scale bars represent 100 µM. Data shown as mean ± SD. *P < 0.05.

Through its caveolin scaffolding domain, CAV-1 functionally represses cell proliferation via (receptor) tyrosine kinase binding, such as platelet-derived growth factor receptor, which revealed that CAV-1/PDGFR regulation are the upstream events of cell proliferation^13^. Therefore, we further investigated the influence of Zed on the CAV-1/PDGF pathway in HBSMCs. qPCR results revealed that Zed increased the relative mRNA levels of *CAV1* compared to those in cells only stimulated with PDGF (Fig. 4a). Moreover, Zed significantly enhanced the expression of CAV-1 protein in HBSMCs, as determined by western blot analysis (Fig. 4b, c). In contrast, Zed relatively suppressed p-PDGFR-β production (Fig. 4b, d). For further verification, we costained CAV-1 and PDGFR-β in HBSMCs by using confocal microscopy and found that the colocalization of CAV-1 and PDGFR-β increased significantly (Fig. 4e). Overall, these findings are in line with the proteomic analysis, suggesting that Zed could modulate CAV-1/PDGFR-β binding upon PDGF-BB stimulation.

### Zedoarondiol inhibits PDGF-BB-induced activation of the downstream signalling pathway of CAV-1/PDGFRβ in HBSMCs

**Figure 5:**
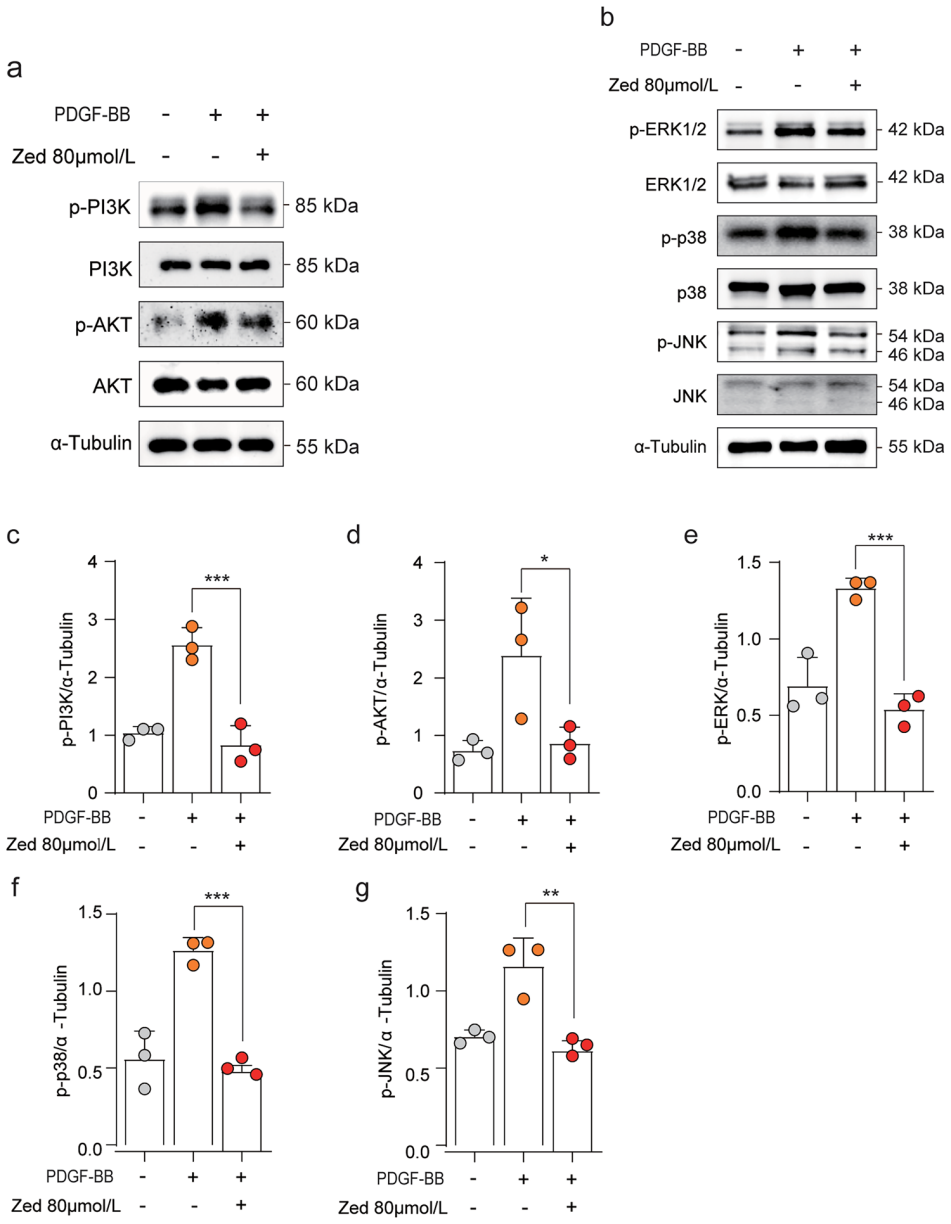
Zedoarondiol down-regulates the downstream signalling pathway of MAPK, PI3K/AKT in HBSMCs cells. (**a**, **b**) Western blot analysis of PI3K/AKT, MAPK in HBSMCs treated as described in Fig. 1d. (n = 3 per group). (**c**–**g**) Quantitative analysis of protein expression was shown. Data shown as mean ± SD. *P < 0.05.

We next investigated the molecular mechanisms underlying the effects of Zed. Studies have shown that the MAPK and PI3K/AKT signalling pathways are the main mitogenic pathways involved in the proliferation of ASMCs^14,16^. Therefore, we examined the effect of Zed on the expression of the downstream signalling pathway of CAV-1/PDGFR-β in PDGF‐BB‐stimulated HBSMCs. We found that Zed inhibited p-PI3K, p-AKT, p-ERK, p-P38 and p-JNK expression at the protein level, as shown by western blotting (Fig. 5a–g). The above proteins can also regulate cell proliferation but showed a downwards expression trend, suggesting that Zed could effectively inhibit HBSMC proliferation.

### CAV-1 knockdown abrogates the inhibitory effect of Zed in HBSMCs

**Figure 6:**
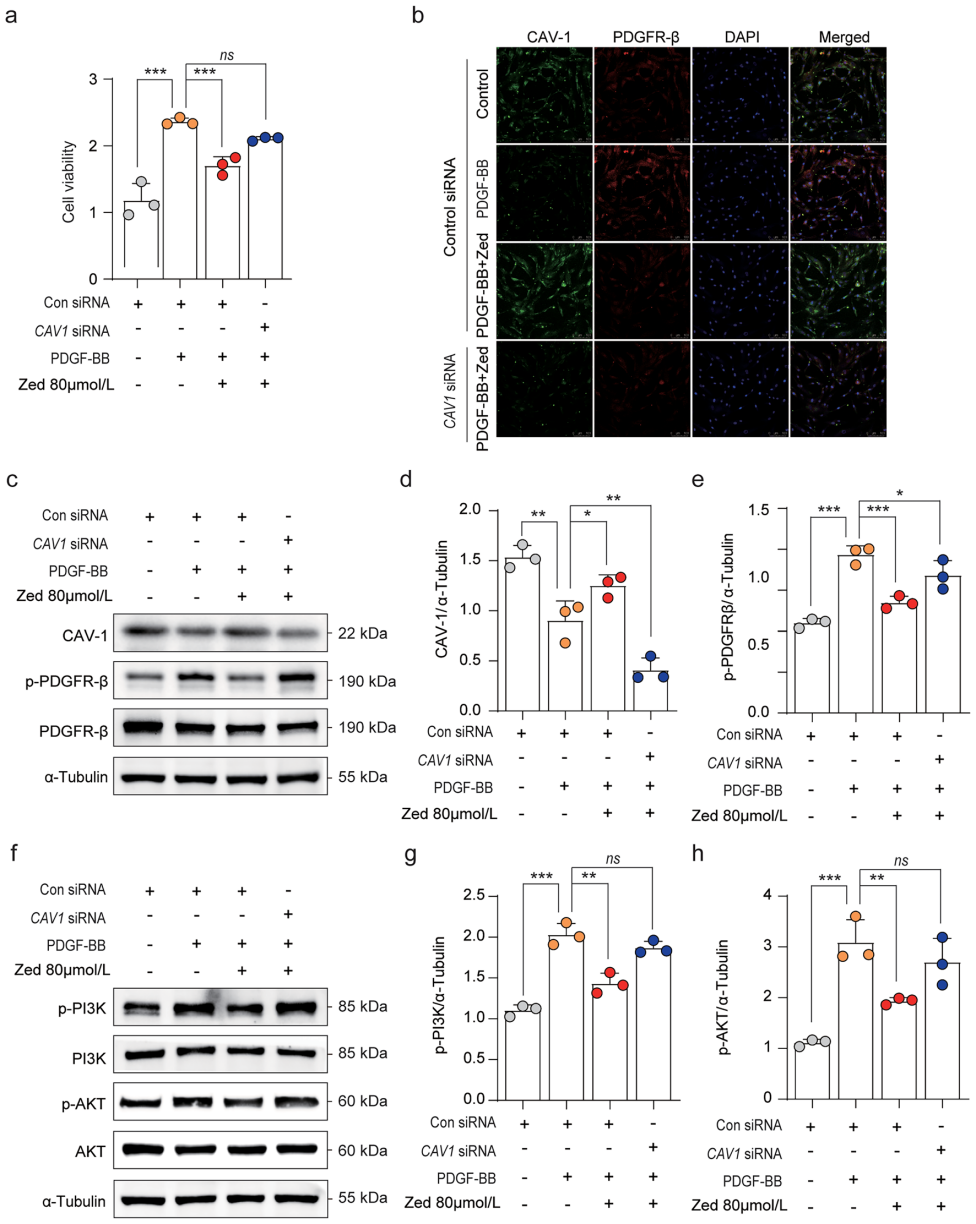
*CAV1* knockdown reverses the inhibitory effect of zedoarondiol in HBSMCs. (**a**) The cell viability was tested using a CCK-8 assay. HBSMCs were treated with *CAV1* siRNA (50 nM), then pre-treated with Zed for 24 h, next was co-treated with Zed and PDGF for 24 h (n = 3 per group). (**b**) The colocalization of CAV-1 (green) and PDGFR-β (red) in HBSMCs treated as in (a) (n = 6 per group). (**c**, **f**) Western blot analysis of CAV-1/PDGFR-β, PI3K/AKT in HBSMCs. HBSMCs were treated as described (**a**). (**d**, **e**, **g**, **h**) Quantitative analysis of protein expression was shown. Data shown as mean ± SD. *P < 0.05.

To further verify whether CAV-1 expression is required for Zed’s inhibitory effects on HBSMC proliferation, we next employed an siRNA knockdown strategy to reduce CAV-1 expression. First, we explored the best transfection concentration and time of siRNA. Initial experiments indicated that *CAV1*-siRNA-Sequence C transfection into HBSMCs can maximally downregulate the expression of CAV-1 at both the mRNA and protein levels (Supplementary Fig. 1). Next, a CCK-8 assay was conducted to determine the effect of Zed on HBSMC proliferation after *CAV1* knockdown. As shown in Fig. 6a, *CAV1* knockdown partly reversed the inhibitory effects of Zed on HBSMC proliferation. We also found that Zed promoted the colocalization of CAV-1 with PDGFR-β in HBSMCs induced by PDGF-BB. However, *CAV1* knockdown significantly suppressed the colocalization of CAV-1 and PDGFR-β and the expression of CAV-1, as determined by immunofluorescence and western blot analysis (Fig. 6b–e). Moreover, knockdown of *CAV1* enhances the expression of phosphorylated proteins, which contribute to cellular proliferation as a result of Zed-mediated inhibition (Fig. 6f–h). Thus, these results collectively indicate that the inhibitory effect of Zed depends on the negative regulation of the proliferation signalling pathway by CAV-1.

## Discussion

Pharmacologically suppressing ASMC proliferation is an important strategy to prevent and alleviate airway remodelling and has important significance in the prevention and treatment of airway-related diseases. Zed, a natural compound isolated from the herb drug *Zedoariae rhizome*, is a new type of zedoary guaiane sesquiterpene compound. In the current study, we explored the effects of Zed on PDGF-BB-mediated HBSMC proliferation and investigated the possible molecular mechanisms. For the first time, we demonstrated that Zed effectively inhibits HBSMC proliferation, causes HBSMC arrest in the G1 phase and induces apoptosis. Moreover, we found that Zed treatment inhibits PDGF-BB-induced activation of the downstream PI3K/AKT and MAPK signalling pathways in HBSMCs. More importantly, our results suggest that the inhibitory effects of Zed depend on the negative regulation of the proliferation signalling pathway by CAV-1. The results of this study showed that Zed could inhibit the PDGF-BB induced hyperproliferation of HBSMCs, promote CAV-1 expression, and competitively inhibit PDGFR-β and the activation of PI3K/AKT signalling pathways that regulate proliferation downstream. Silencing *CAV1* by siRNA transfection not only reduced CAV-1 expression, but also intervened with Zed on the basis of proliferation model, which could not promote CAV-1 expression, and the state of cell hyperproliferation did not improve. The downstream PI3K/AKT signalling pathways that regulate cell proliferation were highly expressed. In conclusion, CAV-1/PDGF pathway plays an important role in inhibiting the proliferation of HBSMCs. Zed competitively inhibits PDGFR-β by promoting CAV-1 expression and MAPK, PI3K/AKT signalling pathway activation, inhibit airway smooth muscle cell proliferation. When* CAV1* was silenced, the effect of Zed on inhibiting the proliferation of HBSMCs was weakened. Thus, our study unravels a previously unappreciated mechanism of action of Zed, indicating that it might be a candidate drug for the treatment of airway-related diseases. PDGF, as the main mitogen of ASMCs, is a key molecule in inducing ASMC proliferation. Previous studies have demonstrated that PDGF‐BB induces ASMC phenotypic modulation^21,22^. Therefore, PDGF‐BB is typically used to induce ASMCs in vitro. Its isotype ligand binds to and activates two structurally related RTK (α receptor and β receptor) dimer complexes that play cellular roles, leading to cell growth, chemotaxis, actin recombination, and preventing apoptosis^23^. PDGFR is a type of RTK. PDGF-PDGFR binding induces a complex signalling network composed of many downstream signalling molecules to participate in the proliferation process of ASMCs^9,18,24^. PDGFR-β is the main PDGF receptor on the surface of HBSMCs, and its activity is controlled by multiple factors at both the intracellular and extracellular levels. In this study, PDGF-BB was used to construct an abnormal proliferation model of HBSMCs and to simulate the excessive proliferation of ASMCs that cause airway remodelling in vitro.

**Figure 7:**
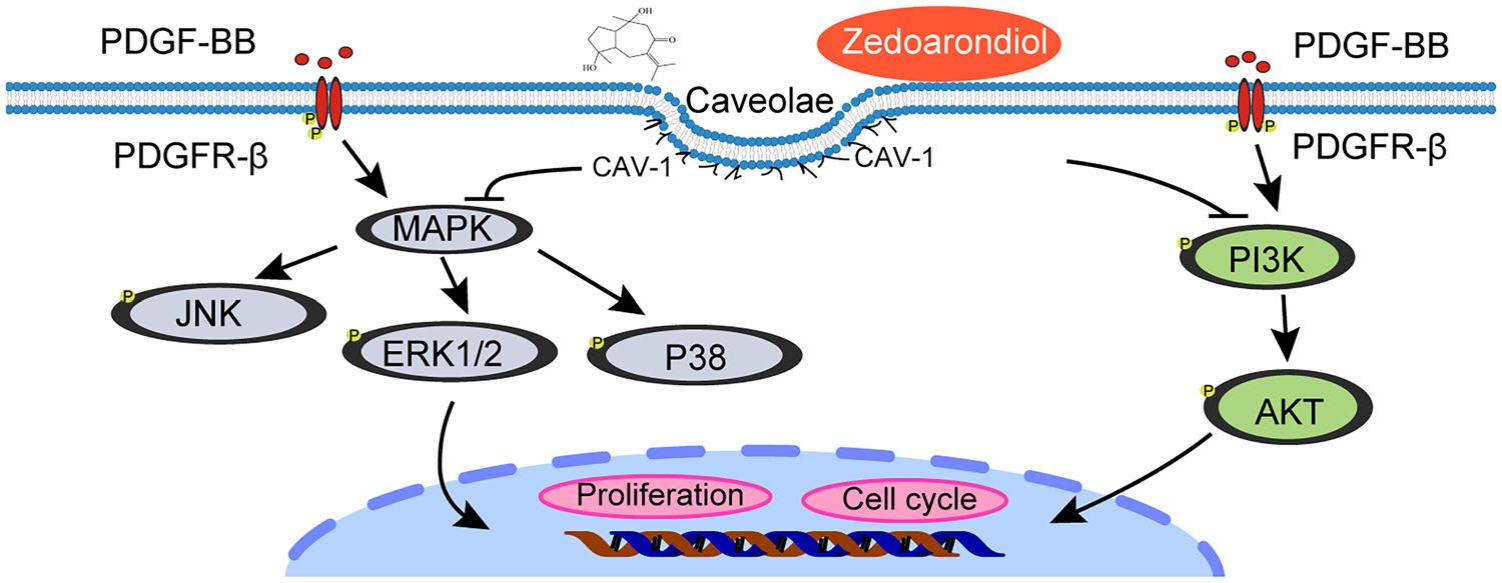
The mechanism of zedoarondiol regulated HBSMCs proliferation. Working model depicting zedoarondiol promotes CAV-1 expression to regulate CAV-1-mediated negative regulation of proliferation signalling pathway, consequently inhibiting HBSMCs proliferation.

It has been well documented that CAV-1 plays an important role in regulating growth factor PDGF-induced signal transduction and cell proliferation^16,25,26^. A remarkable repertoire of functions of CAV-1 has been identified, and these extend to functions related to asthma and chronic inflammatory respiratory diseases^27–29^. Caveolin scaffold domains (CSDs) in the N-terminal region of CAV-1 play a major role in its function. The CAV-1 signalling molecule can inhibit downstream signals through its interaction with CSDs^28^. Research has revealed that CAV-1 functionally represses ASMC proliferation via its caveolin scaffolding domain that binds to RTKs, such as platelet-derived growth factor receptor and epidermal growth factor receptor^30^. In airway smooth muscle, RTK associates reversibly with caveolae and CAV-1, as mitogen stimulation induces the release of caveolin-1 from the activated RTK, which facilitates RTK trafficking to the non-caveolae membrane^23,31^. RTK combines with PDGF to form a dimer and undergoes self-phosphorylation. Phosphorylated RTK activates downstream signalling pathways through multiple pathways^32^. The three main signalling pathways of MAPK, the ERK, JNK, and p38 and PI3K/AKT signalling pathways, have been recognized as the main mitogenic pathways involved in the proliferation of ASMCs^33–37^. Intriguingly, proteomic analysis in our study revealed that CAV-1//PDGF and its downstream signalling pathways, which participate in cell proliferation, might play critical roles in the inhibitory effect of Zed on cell proliferation. Therefore, we further investigated the influence of Zed on CAV-1/PDGF and its downstream signalling pathways in HBSMCs. Mechanistically, as illustrated in Fig. 7, our results showed that Zed treatment upregulated CAV-1 expression, increased the colocalization of CAV-1 and PDGFR-β, and inhibited the downstream MAPK and PI3K/AKT signalling pathways in HBSMCs.

In addition, to verify the importance of the CAV-1/PDGF pathway in the effect of Zed, an siRNA knockdown strategy was employed to reduce CAV-1 expression. A previous study demonstrated that the use of methyl-β-cyclodextrin (MβCD)-modified caveolae protein or knockout of the *CAV1* gene induced excess ASMC proliferation. Genetic ablation of CAV-1 most likely affects the cell cycle process of G1/S transformation through the disturbance of AKT signal transduction^30^. Consistent with previous studies^38^, si-*CAV1* reversed the inhibitory effects of insulin-like growth factor 1 (IGF-1) on HBSMC proliferation and the expression of p-PI3K and p-AKT, which suggests that Zed suppresses cell proliferation in a CAV-1-dependent manner. This study suggests that Zed is a natural CAV-1 agonist.

Taken together, our findings provide strong evidence for the novel pharmacological effect of Zed: it inhibits HBSMC proliferation by promoting CAV-1 expression to block MAPK and PI3K/AKT activation in vitro. These findings highlight the potential mechanism of Zed in the treatment of airway remodelling and related diseases, pending further clinical studies. Due to time constraints, cell experiments are only carried out in vitro at present. Therefore, animal models of asthma will be further used for in vivo animal experiments in the future to continuously improve the mechanism of Zed in the treatment of airway remodelling in asthma.

### Supplementary Information

Supplementary Information.

## Data Availability

The datasets used and/or analysed during the current study available from the corresponding author on reasonable request.
