# Supplementary material for: Zedoarondiol inhibits human bronchial smooth muscle cell proliferation through the CAV-1/PDGF signalling pathway
Source: Sci Rep. 2024 Jun 7;14:13145. doi: 10.1038/s41598-024-63970-4 (PMC11161633; doi:10.1038/s41598-024-63970-4)

Supplemental Table. 1 The scrambled siRNA sequenced used for *CAV1* knockdown.

Sequences	Forward	Reverse
A	GACGUGGUCAAGAUUGACUUUTT	AAAGUCAAUCUUGACCACGUCTT
B	CCACCUUCACUGUGACGAAAUTT	AUUUCGUCACAGUGAAGGUGGTT
C	AGACGAGCUGAGCGAGAAGCATT	UGCUUCUCGCUCAGCUCGUCUTT

Supplemental Table. 2 Antibody suppliers, catalog number and molecular weight.

Antibody	Supplier	Catalog number	Molecular weight(kDa)
Caveolin-1	Proteintech	66067-1-Ig	22
p-PDGFRβ	Cell Signaling Technology	#3166	190
PDGFRβ	Cell Signaling Technology	#3169	190
p-PI3K	Abcam	ab182651	84
PI3K	Proteintech	60225-1-Ig	85
p-AKT	Proteintech	66444-1-Ig	60-62
AKT	Proteintech	10176-2-AP	56
p-ERK1/2	Cell Signaling Technology	#4370	42,44
ERK1/2	Proteintech	67170-1- Ig	38-43
p-p38	Cell Signaling Technology	#9216	43
p38	Proteintech	14064-1-AP	35,41
p-JNK	Proteintech	80024-1-RR	37-45
JNK	Proteintech	66210-1- Ig	44-48,50-55
α-Tubulin	Proteintech	11224-1-AP	50
Rabbit IgG H&L	Abcam	ab8245	N/A
Mouse IgG H&L	Abcam	ab6728	N/A
Goat Anti-Rabbit IgG H&L / AF594	Bioss	bs-0295G-AF594	N/A
Goat Anti-Mouse IgG H&L / FITC	Bioss	bs-0296G-FITC	N/A

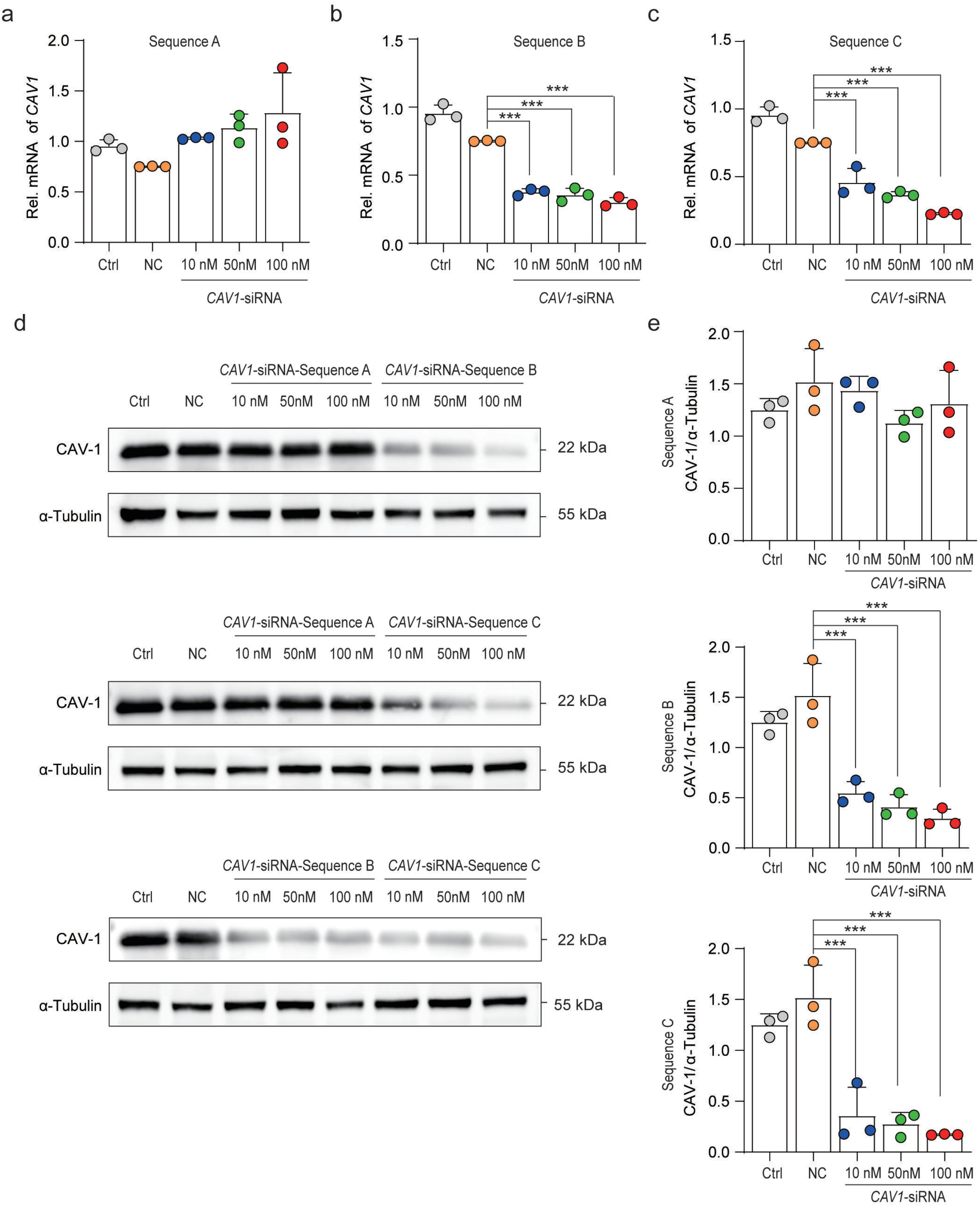
a
Sequence A
Rel. mRNA of CAV1
2.0
1.5
1.0
0.5
0.0
Ctrl
NC
10 nM
50nM
100 nM
CAV1-siRNA
b
Sequence B
Rel. mRNA of CAV1
1.5
1.0
0.5
0.0
***
***
***
Ctrl
NC
10 nM
50nM
100 nM
CAV1-siRNA
c
Sequence C
Rel. mRNA of CAV1
1.5
1.0
0.5
0.0
***
***
***
Ctrl
NC
10 nM
50nM
100 nM
CAV1-siRNA
d
CAV1-siRNA-Sequence A
CAV1-siRNA-Sequence B
Ctrl
NC
10 nM
50nM
100 nM
10 nM
50nM
100 nM
CAV-1
22 kDa
α-Tubulin
55 kDa
CAV1-siRNA-Sequence A
CAV1-siRNA-Sequence C
Ctrl
NC
10 nM
50nM
100 nM
10 nM
50nM
100 nM
CAV-1
22 kDa
α-Tubulin
55 kDa
CAV1-siRNA-Sequence B
CAV1-siRNA-Sequence C
Ctrl
NC
10 nM
50nM
100 nM
10 nM
50nM
100 nM
CAV-1
22 kDa
α-Tubulin
55 kDa
e
Sequence A
CAV-1/α-Tubulin
2.0
1.5
1.0
0.5
0.0
Ctrl
NC
10 nM
50nM
100 nM
CAV1-siRNA
Sequence B
CAV-1/α-Tubulin
***
***
***
2.0
1.5
1.0
0.5
0.0
Ctrl
NC
10 nM
50nM
100 nM
CAV1-siRNA
Sequence C
CAV-1/α-Tubulin
***
***
***
2.0
1.5
1.0
0.5
0.0
Ctrl
NC
10 nM
50nM
100 nM
CAV1-siRNA

Supplementary Fig 1. Effects of different *CAV1* siRNA base sequences and concentrations on *CAV1* expression. (a-c) qPCR analysis of CAV1 mRNA expression in HBSMCs treated as described above (n = 3 per group). (d) Western blot analysis of CAV-1 in HBSMCs. (e) Quantitative analysis of protein expression was shown. Data shown as mean ± SD. *$P < 0.05$.

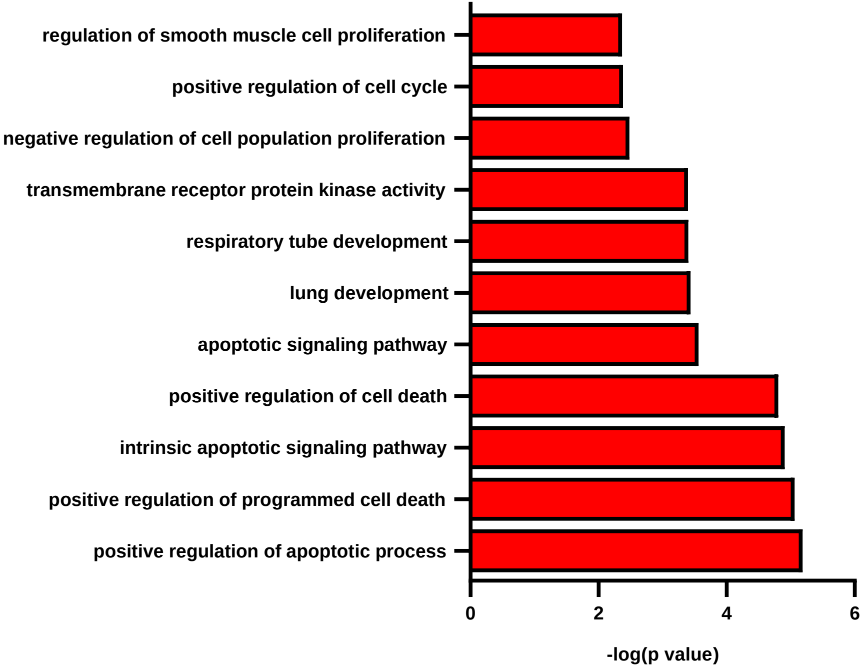
regulation of smooth muscle cell proliferation
positive regulation of cell cycle
negative regulation of cell population proliferation
transmembrane receptor protein kinase activity
respiratory tube development
lung development
apoptotic signaling pathway
positive regulation of cell death
intrinsic apoptotic signaling pathway
positive regulation of programmed cell death
positive regulation of apoptotic process
0
2
4
6
-log(p value)

Supplementary Fig 2. GO pathway enrichment analysis (BP: biological process) based on significantly changed proteins. Representative pathways are shown.

Cropped blots in figure 4

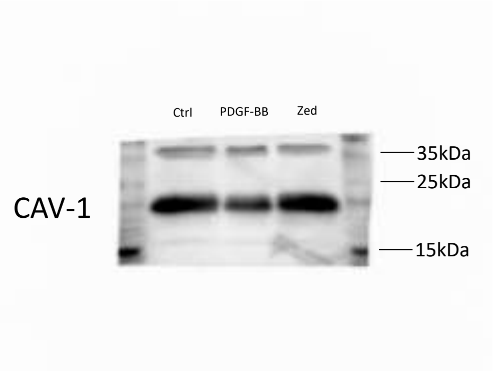

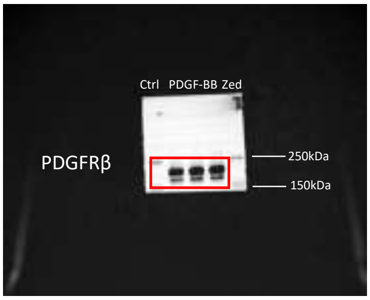

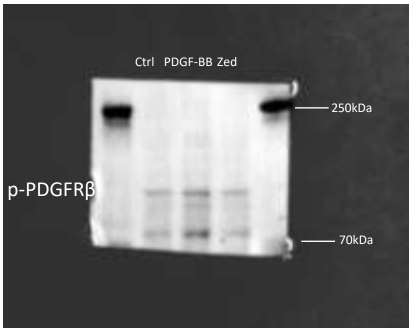

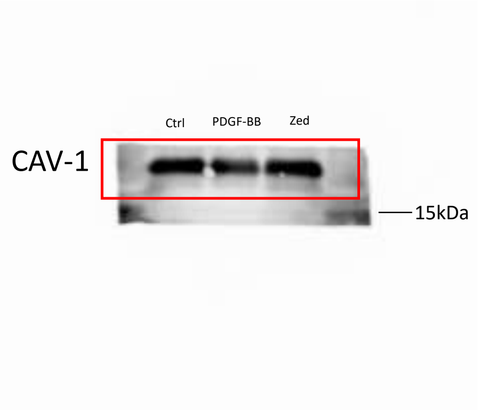

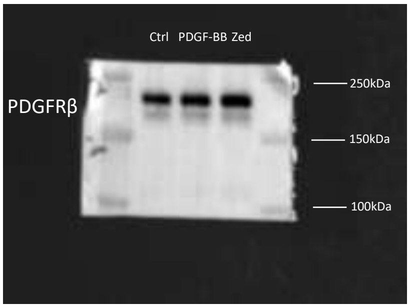

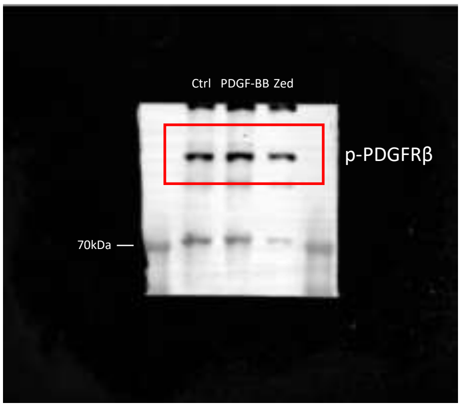

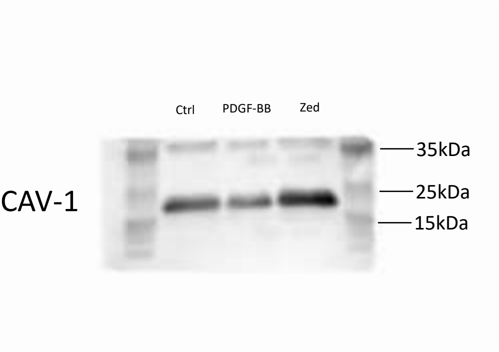

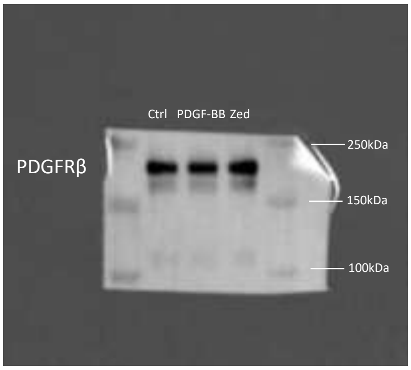

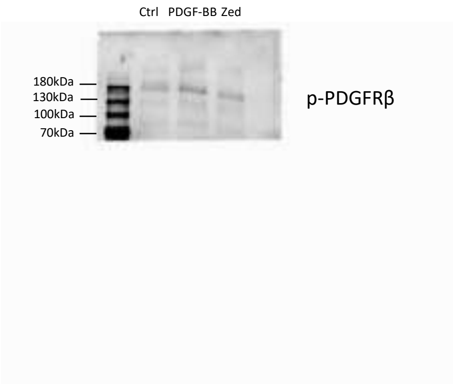

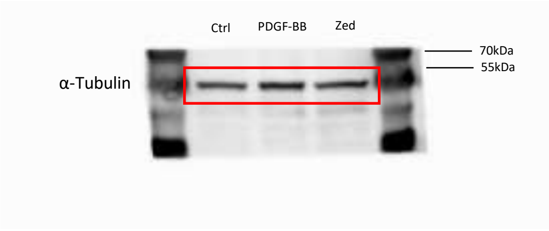

Cropped blots in figure 5

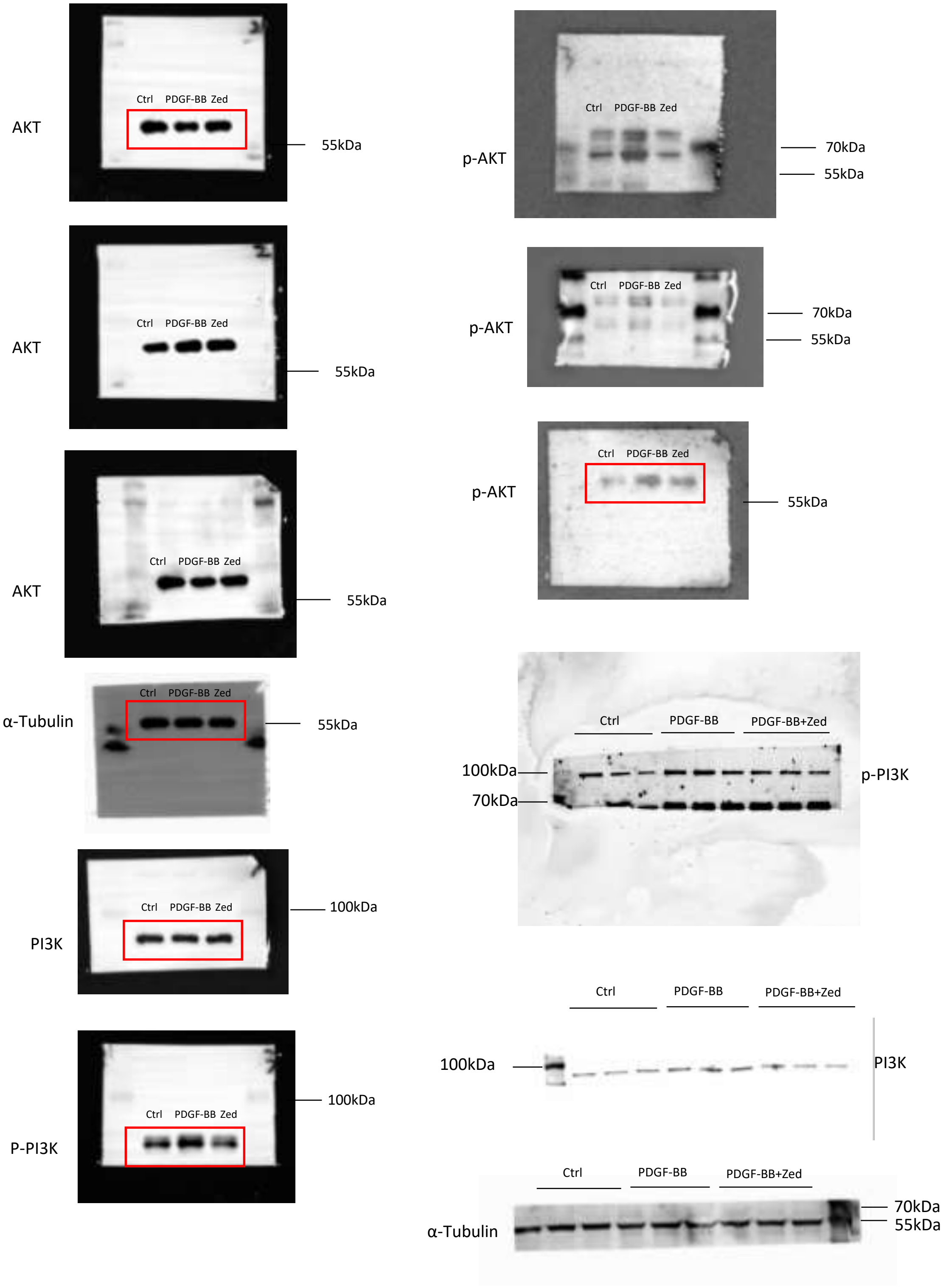

Cropped blots in figure 5

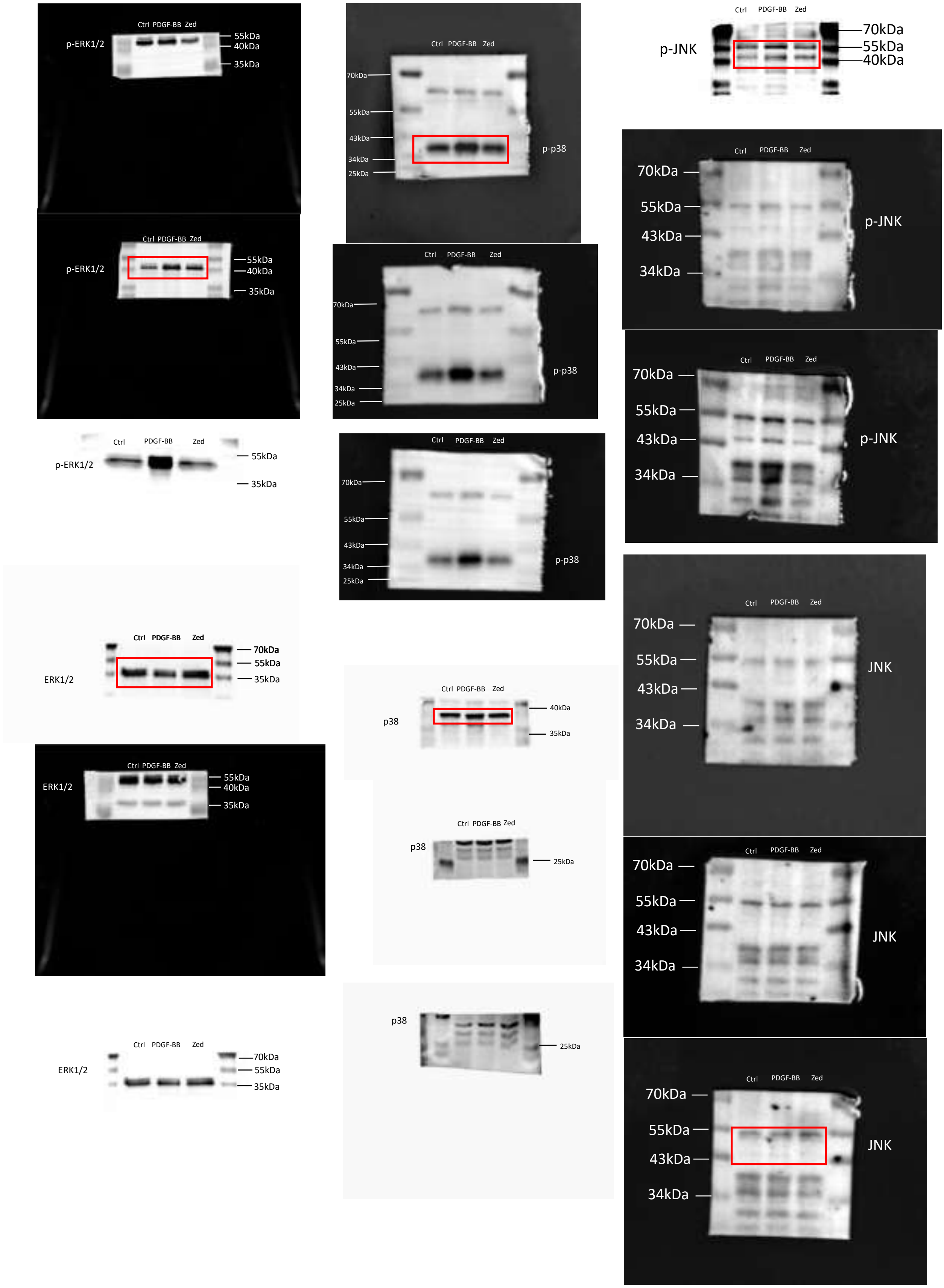

Cropped blots in Optimal sequence and concentration for transfection

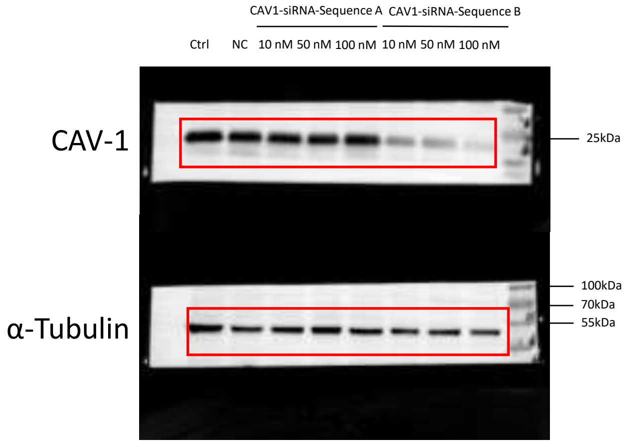

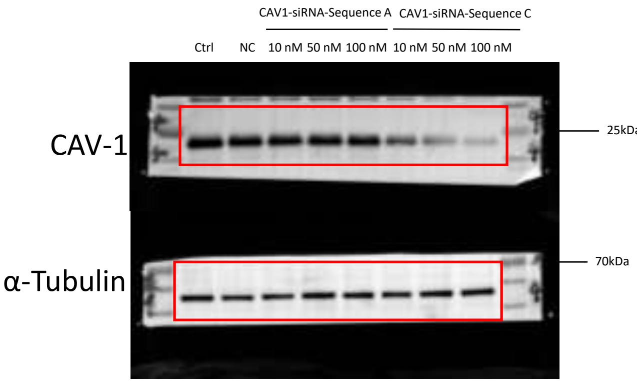

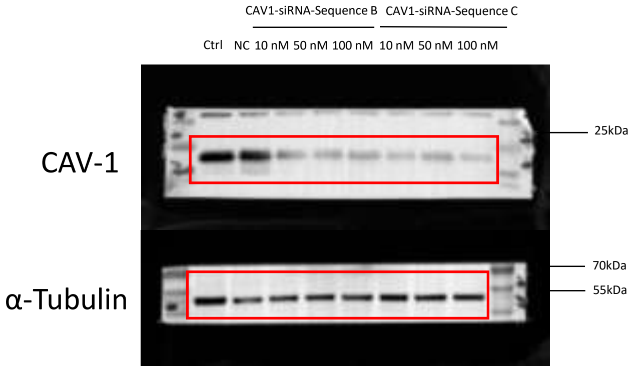

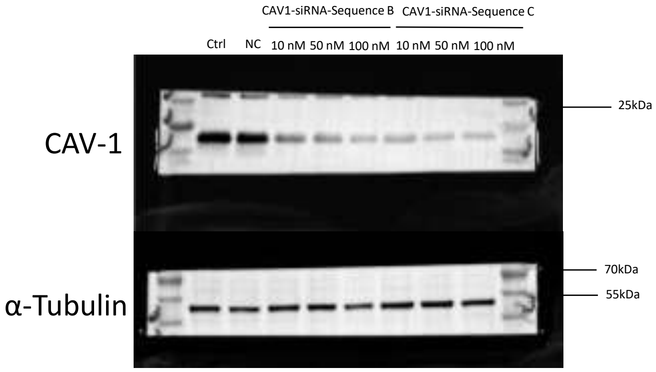

Cropped blots in figure 6

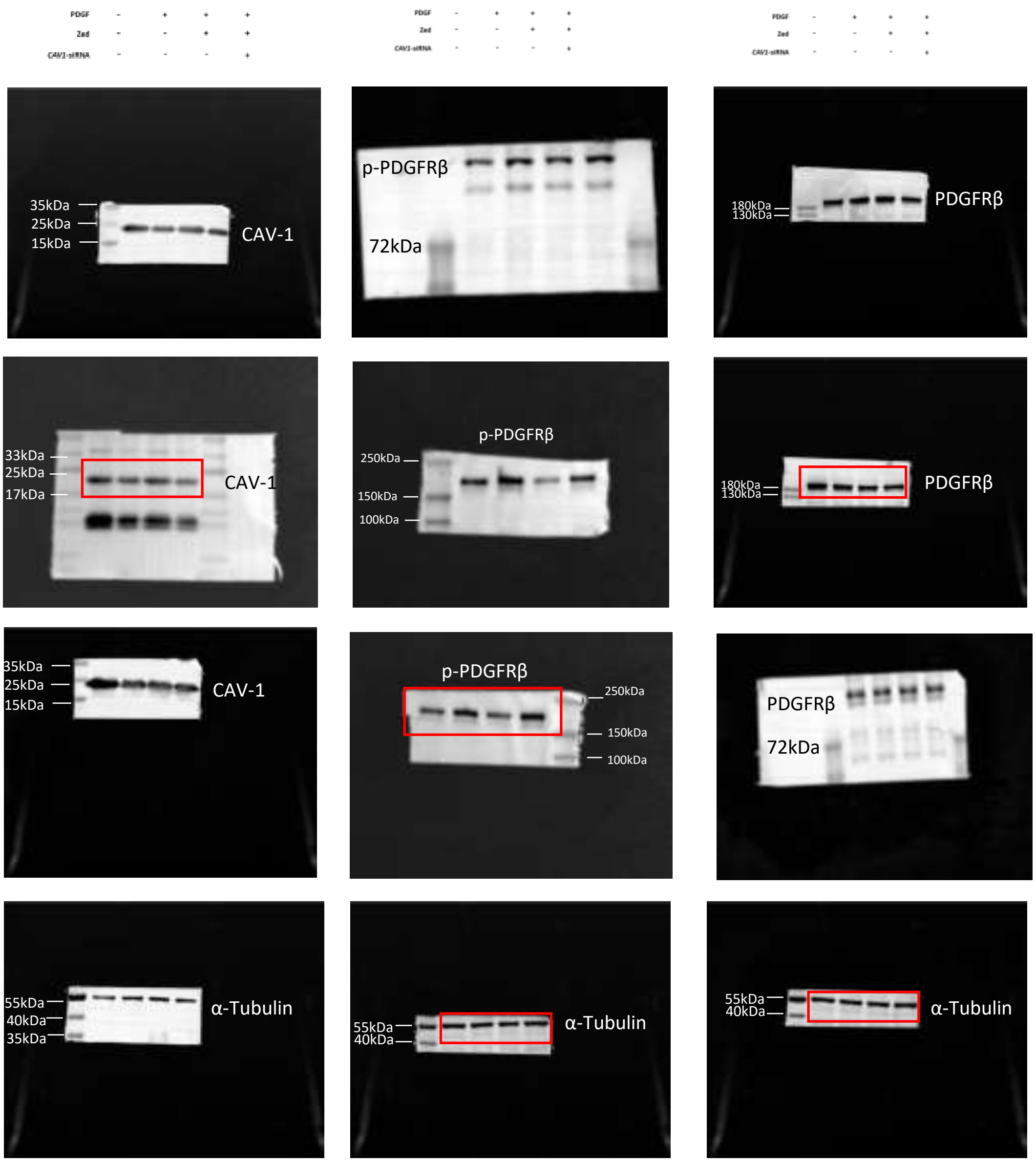

Cropped blots in figure 6

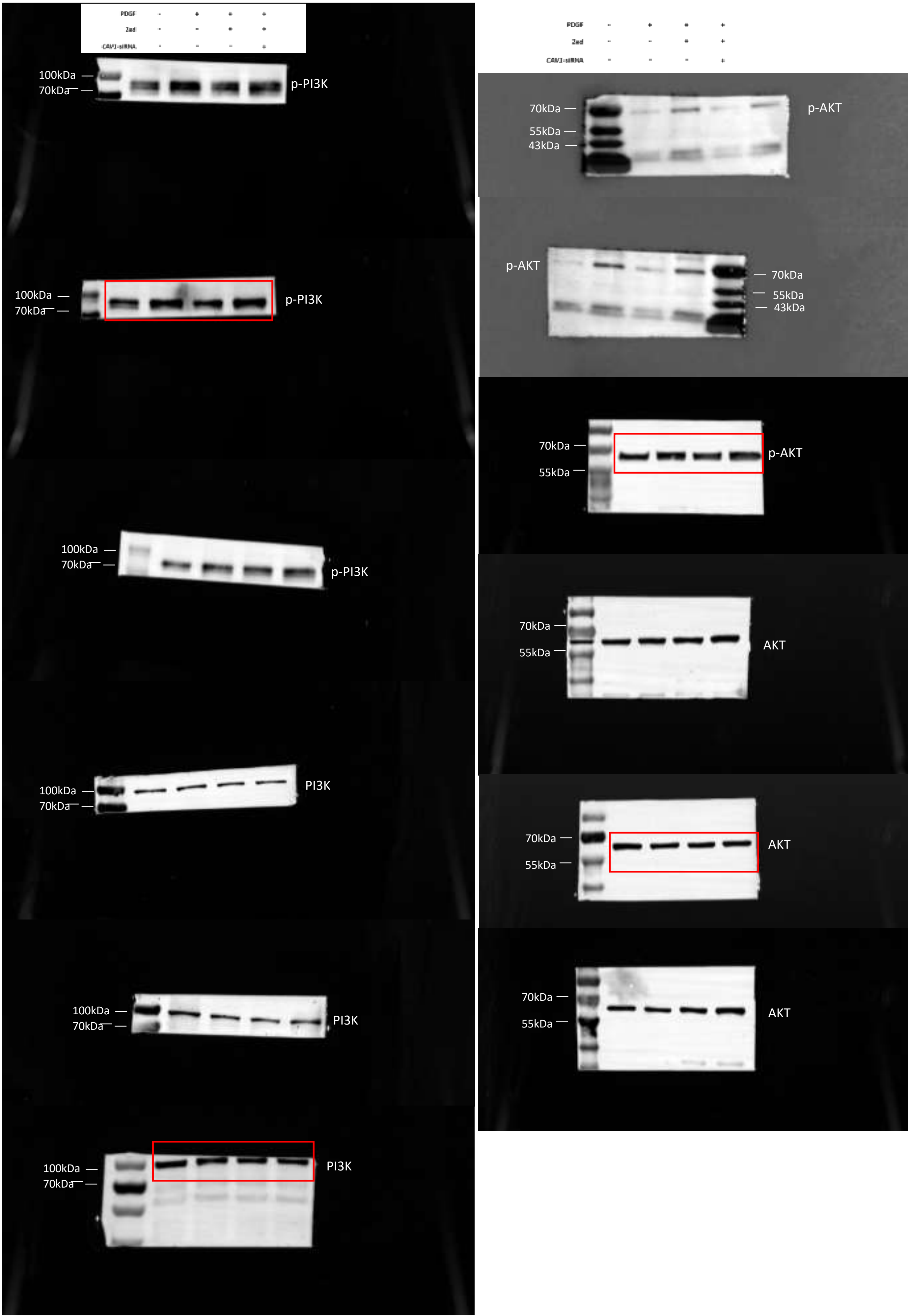

Supplement: Supplementary file 1 — Supplementary Information. [file 41598_2024_63970_MOESM1_ESM.pdf]
